# Role of the intestinal microbiome and microbial-derived metabolites in immune checkpoint blockade immunotherapy of cancer

**DOI:** 10.1186/s13073-021-00923-w

**Published:** 2021-06-23

**Authors:** Eiko Hayase, Robert R. Jenq

**Affiliations:** 1 Department of Genomic Medicine, The University of Texas MD Anderson Cancer Center, Houston, TX USA; 2 Department of Stem Cell Transplant and Cellular Therapy, The University of Texas MD Anderson Cancer Center, Houston, TX USA; 3 CPRIT Scholar in Cancer Research, Houston, TX USA

**Keywords:** Intestinal microbiome, Metabolites, Immune checkpoint inhibitors

## Abstract

Immune checkpoint inhibitors (ICIs) are monoclonal antibodies that block immune inhibitory pathways. Administration of ICIs augments T cell-mediated immune responses against tumor, resulting in improved overall survival in cancer patients. It has emerged that the intestinal microbiome can modulate responses to ICIs via the host immune system and that the use of antibiotics can lead to reduced efficacy of ICIs. Recently, reports that fecal microbiota transplantation can lead to ICI therapy responses in patients previously refractory to therapy suggest that targeting the microbiome may be a viable strategy to reprogram the tumor microenvironment and augment ICI therapy. Intestinal microbial metabolites may also be linked to response rates to ICIs. In addition to response rates, certain toxicities that can arise during ICI therapy have also been found to be associated with the intestinal microbiome, including in particular colitis. A key mechanistic question is how certain microbes can enhance anti-tumor responses or, alternatively, predispose to ICI-associated colitis. Evidence has emerged that the intestinal microbiome can modulate outcomes to ICI therapies via two major mechanisms, including those that are antigen-specific and those that are antigen-independent. Antigen-specific mechanisms occur when epitopes are shared between microbial and tumor antigens that could enhance, or, alternatively, reduce anti-tumor immune responses via cross-reactive adaptive immune cells. Antigen-independent mechanisms include modulation of responses to ICIs by engaging innate and/or adaptive immune cells. To establish microbiome-based biomarkers of outcomes and specifically modulate the intestinal microbiome to enhance efficacy of ICIs in cancer immunotherapy, further prospective interventional studies will be required.

## Background

Immune checkpoint inhibitors (ICIs) are monoclonal antibodies that antagonize immune inhibitory pathways known as checkpoints. The most widely used ICIs are anti-cytotoxic T lymphocyte antigen-4 (CTLA-4), anti-programmed death protein-1 (PD-1), and anti-programmed death-ligand 1 (PD-L1) antibodies [1–3]. These drugs are not thought to be directly tumoricidal, but rather indirectly mediate anti-tumor effects by inducing T cell activation mechanisms [4]. Development of ICI therapies has been a major clinical advance, and these are now an important tool in the treatment of cancer. Administration of ICIs augments T cell-mediated immune responses against tumor cells, and these treatments have been found to improve overall survival in patients with multiple cancer types [3, 5]. Responses to these therapies, however, are heterogeneous and not always durable [6, 7]. In addition, an important limitation of treatment with ICIs is the incidence of immune-related adverse events, including, in particular, inflammatory colitis [8]. For these reasons, researchers and clinicians have sought to identify biomarkers that can serve as predictors of response and toxicity with ICIs. To date, readouts including tumor mutational load which is a measure of the number of mutations in a cancer, T cell gene expression profiling, and the presence of tumor-infiltrating lymphocytes have all show promise as biomarkers for ICI therapy [9, 10]. In addition, it has recently emerged that differences in the composition of the intestinal microbiome between patients could explain some of the variability in therapeutic responses and immune-related gut adverse event such as colitis [11–13].

The human body harbors trillions of resident microbes that comprise the microbiome, which plays a vital role in many aspects of human health and disease. The intestinal microbiome plays a particularly important role in shaping systemic immune responses [14, 15]. In the context of cancer, it has been demonstrated that the composition of the intestinal microbiome can modulate the efficacy of chemotherapeutic agents by modulating the degree of immune activation [16–18]. Identifying associations between the microbiome and clinical outcomes remains a substantial challenge, due to considerable heterogeneity in the microbiome of both patients and healthy individuals, as well as the complexity of the microbiome itself. Despite this, researchers have recently identified a key role of the intestinal microbiome in treatement with ICIs in both preclinical mouse models and retrospective studies in cancer patients. Here, we focus on recent insights into the intestinal microbiome and microbiome-derived metabolites associated with clinical responses and toxicities in ICI therapy.

### Intestinal microbiome and responses to ICI therapy

Preclinical models have shown that the intestinal microbiome composition regulates efficacy of cancer therapies such as chemotherapy [16–18]. Interestingly, the microbiome was found to particularly modulate immune effects of chemotherapy. T cells, especially cytotoxic T cells, are an important immune cell population that can target tumor cells, and increased T cell infiltration into tumors has been found to be associated with favorable patient outcomes in various cancers [19–22]. Previous microbiome studies have identified specific microbes that were associated with better anti-tumor T cell responses following cancer therapy [23]. A similar search for specific microbes associated with the efficacy of ICIs has been a focus of several recent studies.

Pre-clinical mouse models have been a critically important tool for studying the potential effects of microbes on ICIs. By comparing the anti-tumor efficacy of anti-CTLA-4 antibody in mice maintained under conditions in specific-pathogen-free (SPF) that are free of particular pathogens, and germ-free (GF) that harbor no living microorganisms, a pioneering study found that anti-CTLA-4 antibody treatment controlled tumor progression in SPF but not in GF animals [24]. The anti-tumor effects of anti-CTLA-4 antibody were similarly compromised in mice treated with a broad-spectrum antibiotic cocktail to eliminate the intestinal microbiome [24]. This indicated that an intact commensal microbiome is required to develop effective anti-tumor responses following treatment with anti-CTLA-4 antibody. Recolonization experiments of previously antibiotic-treated SPF mice or GF mice with specific bacterial isolates from the small intestinal mucosa of SPF mice also showed that introduction of *Bacteroides fragilis*, *Bacteroides thetaiotaomicron*, *Burkholderia cepacia*, or the combination of *B. fragilis* and *B. cepacia* led to restoration of anti-CTLA-4 antibody-mediated anti-tumor responses [24]. Another pivotal study began with the observation that the efficacy of anti-PD-L1 antibody against melanoma was different in mice derived from two different animal facilities, Jackson Laboratory and Taconic Farms. Profiling of the intestinal bacterial composition of these mice using 16S ribosomal RNA (16S rRNA) analysis found that intestinal *Bifidobacterium* was associated with superior anti-tumor effects of anti-PD-L1 antibody [25]. In addition, oral administration of *Bifidobacterium* improved anti-tumor effects of ICIs by augmenting dendritic cell function and activating cytotoxic CD8^+^ T cells. Heat inactivation of *Bifidobacterium* before oral administration abrogated the effects on tumor and T cell responses, suggesting that specific live commensal bacteria may modulate responses to ICIs against tumor cells. Thus, these studies have successfully demonstrated that mouse-derived commensal bacteria can support anti-tumor effects in ICI therapy.

Subsequent studies focused on the human-derived commensal microbiome to identify specific bacteria that can improve anti-tumor efficacy. Metagenomic studies of patient stool samples have revealed significant associations between the intestinal microbiome and clinical responses in different solid cancers. In a study of metastatic melanoma patients, Chaput et al. showed that *Faecalibacterium prausnitzii* and *Gemmiger formicilis* were associated with a positive response to anti-CTLA-4 antibody whereas Bacteroides were associated with poor response [12]. Gopalakrishnan et al. found significantly higher α-diversity and a higher relative abundance of Ruminococcaceae family members in melanoma patients responding to anti-PD-1 antibody [26]. Routy et al. found associations between clinical responses to ICIs and increased relative abundances of *Akkermansia muciniphila* and *Enterococcus hirae* in patients with advanced non-small cell lung cancers or urothelial carcinomas [27]. Matson et al. found that *Bifidobacterium longum*, *Collinsella aerofaciens*, and *Enterococcus faecium* were more abundant in melanoma patients responding to anti-PD-1 antibody whereas *Ruminococcus obeum* and *Roseburia intestinalis* were more abundant in non-responding patients [28]. GF mice or antibiotic-treated mice that received fecal microbiota transplantation (FMT) from cancer patients who responded to ICIs showed improved anti-tumor effects [26–28]. Using a different approach that examined immune responses to the introduction of bacteria in GF mice, Tanoue et al. found that a consortium of 11 bacterial strains isolated from healthy human donor stool could robustly induce interferon-γ (IFNγ)-producing CD8^+^ T cells in the intestine [29]. These bacterial strains also enhanced ICI-mediated anti-tumor effects in a CD8^+^ T cell-dependent manner in a mouse model. Recently, the potential role of B cells within tertiary lymphoid structures in the response to ICIs was also reported in patients with melanoma and renal cell carcinoma [30]. However, potential associations between B cell responses in ICI therapy and the intestinal microbiome have yet to be extensively examined.

Altogether, these studies indicate that the intestinal microbiome in both mice and humans can be important modulators of tumor responses to ICIs via modulation of the host immune system. Lack of consistency, however, in the key bacteria subgroups identified to be associated with tumor response in these studies, has been a major limitation and explanations for this inconsistency have yet to be firmly identified. While sequencing center heterogeneity in technical and computational procedures are known to impact substantially on microbiome readouts [31, 32], this is primarily a challenge only when attempting to combine microbiome results from different groups and should not impact on single-center study results. Other potential explanations include the following: (1) geographical and population differences, (2) microbiome associations that are specific to certain tumor types, and (3) aspects of the microbiome besides bacterial taxonomy that are so far relatively understudied, including genes, metabolites, phages and other viruses, and fungi. Deep sequencing analysis such as shotgun metagenomic sequencing might be helpful to identify bacterial genes as well as bacteriophage and other viruses and fungi associated with response by ICI therapy. Investigating relationships between the commensal microbiome and tumor microbiome might also lead to new insights. Finally, most of the clinical studies to date have been performed on relatively small numbers of patients, and larger numbers of patients are likely needed to rigorously identify associations between the microbiome and a clinical phenotype such as response to ICI therapy which is likely downstream from many contributing factors. This may be similar to other types of high-features studies, such as genome-wide association studies (GWAS), which typically require very large numbers of patients to identify genetic associations with diseases that are heterogeneous in their subtypes and biological underpinnings [33].

There are many environmental and life style factors including dietary habits, smoking, and alcohol consumption that can influence the intestinal microbiome and immune system [34]. Smoking history was reported to associate with improved overall survival and progression-free survival of non-small cell lung cancer patients but the authors concluded that smoking history cannot be recognized as a predictor of ICI efficacy in this systematic review and meta-analysis study because of insufficient numbers of patients not reporting a smoking history [35]. Obesity is associated with the intestinal microbiome and immune phenotypes. A growing number of reports have demonstrated that obesity might be associated with a survival benefit in patients treated with ICIs, though how obesity could induced better clincal reponses remains unknown [36]. In addition, tumor gene modification or immune editing by the intestinal microbiome or environment/life style factors may also explain associations with responses to ICI therapy, as the tumor mutational burden and DNA damage repair gene status are important predictors of efficacy of ICI in cancer patients [37]. Thus, examining not only the intestinal microbiome but also the context of environmental factors will be critical to a better understanding of how to target the microbiome to enhance clinical responses to ICI therapy.

Targeting the intestinal microbiome as a therapeutic strategy to augment ICI is still in its infancy, but progress has been recently reported. Two early single-arm clinical studies of FMT were found to potentially result in improved responses ICI therapy [38, 39]. A phase I clinical trial to assess the safety and feasibility of FMT and reinduction of anti-PD-1 antibody was performed in 10 patients with anti-PD-1-refractory metastatic melanoma [38]. This study included two FMT donors who had previously been treated with anti-PD-1 monotherapy for metastatic melanoma and had achieved a complete response for over 1 year. FMT from complete response donors and reinduction of anti-PD-1 antibody in refractory metastatic melanoma patients was demonstrated to be safe and feasible and in some patients was demonstrated to increase intratumoral immune activity. In another study, it was investigated whether resistance to anti-PD-1 antibody could be overcome be changing the intestinal microbiota in patients with anti-PD-1-refractory melanoma [39]. These patients were treated with anti-PD-1 antibody and FMT obtained from long-term responder melanoma patients. In this study, 3 out of 15 patients experienced objective responses and an additional 3 patients had durable stable disease. These clinical studies suggested that modulation of the intestinal microbiome has the potential to reprogram the tumor microenvironment and overcome resistance to ICI therapy. To definitively identify microbiome parameters that will improve ICI efficacy, including optimal FMT composition and patient microbiome profiles predicted to be likely to respond to FMT, much larger cohorts of patients undergoing this novel therapy will be required.

### Effects of antibiotics on responses to ICI therapy

As described above, preclinical experiments in mice have indicated that use of antibiotics can lead to reduced efficacy of ICIs [11, 24, 27]. In cancer patients, antibiotics are often used for both prophylaxis and curative treatment of a range of potentially life-threating infections that can complicate cancer therapy. They can, however, also select for antibiotic resistance, as well as sometimes produce a profound loss of microbial species found in the intestinal tract. Several retrospective clinical studies in patients with advanced cancers have found that patients who had been recently treated with antibiotics experienced reductions in ICI efficacy [27, 40–42]. Routy et al. reported that patients treated with antibiotics, mainly β-lactams or quinolones, within 2 months before ICI therapy had worse overall survival and progression-free survival. Doresa et al. also reported that patients treated with antibiotics, again mainly β-lactams or quinolones, within 30 days of beginning ICIs had significantly worse overall responses, progression-free survival, and overall survival. Other studies have examined if the timing of antibiotic treatment could distinguish the risk for reduced responses to ICIs, comparing concurrently administered antibiotics to antibiotics administered prior to ICIs [41]. In this study, Pinato et al. found that patients treated with antibiotics, mainly β-lactams, quinolones or macrolides, in the 30 days prior to receiving ICIs were more likely to discontinue ICIs due to disease progression and die of progressive disease while on treatment with ICIs, suggesting that earlier use of antibiotics may be more harmful while antibiotic use while on ICI treatment may be safer. In all these studies, patients were treated with broad-spectrum antibiotics such as β-lactams or quinolones and experienced worse clinical outcomes. However, it remains unclear whether the degree of intestinal microbiome disruption by antibiotics was associated with negative impacts on ICI therapy. Broad-spectrum antibiotics can cause a prolonged disturbance in the gut ecosystem and impair the responses of cytotoxic T cells against cancer [43]. Ahmed et al. retrospectively compared ICI responses in patients who received broad-spectrum antibiotics including 3rd or 4th generation cephalosporins and ciprofloxacin with those who received no broad-spectrum ones within 2 weeks before and after starting ICI therapy and demonstrated that broad-spectrum antibiotics were associated with worse progression-free survival and overall survival in ICI therapy [44]. A recent systematic review and meta-analysis including 48 studies also indicated negative impacts of antibiotic use on overall survival, progression-free survival, response to treatment rate, and progression of disease [45]. Further studies will be required to determine how best to administer antibiotics to patients who will be treated or are undergoing treatment with ICIs including optimal timing, duration, and antibiotic types. For now, it seems reasonable, if possible, to avoid long-term and broad-spectrum antibiotics before starting ICI therapy. In patients who do require antibiotics prior to ICI therapy, an investigational strategy could be to undergo FMT in an attempt to abrogate probable harmful effects of broad-spectrum antibiotics in ICI therapy.

### Effects of intestinal microbial metabolites on responses to ICI therapy

Intestinal microbial metabolites are thought to link the intestinal microbiome to systemic immunity. Researchers have begun to explore the relationship between host, diet, the intestinal microbiome, and microbial-derived metabolites in treatment with ICIs. A major metabolic activity of the intestinal microbiome is the conversion of ingested dietary fiber and mucosal glycans into short-chain fatty acids (SCFAs), which include acetate, propionate, and butyrate [43]. SCFAs can have wide-ranging impacts on host physiology, particularly on immune cells [46], including regulation of intestinal macrophages and promoting gut B cell responses [47]. They are protective in animal models of colitis and colitis-induced colorectal cancer, and can also exert antiproliferative effects on cancer cells [47]. To evaluate for associations between SCFAs and clinical outcomes in cancer patients, Nomura et al. examined fecal and plasma levels of SCFAs in patients with solid tumors treated with anti-PD-1 antibody [48]. They found that higher fecal SCFA concentrations were significantly associated with longer progression-free survival in 52 patients. In the patients with non-small cell lung cancer treated with anti-PD-1 antibody, it was recently shown that fecal SCFAs, especially propionate, were associated with better long-term responses to ICIs [49]. In a clinical study of French and Italian cohorts of patients treated with anti-CTLA-4 antibody, serum SCFA concentrations were evaluated and butyrate concentrations were found to be associated with shorter progression-free survival in the French cohort (n = 40), while propionate concentrations were associated with shorter progression-free survival in the Italian cohort (n = 45) [50].

Tryptophan is an essential amino acid for humans and tryptophan metabolites are known to be bioactive compounds that play important roles in cancer and immune regulation. Most of dietary-derived tryptophan is taken up in the small intestine, but a fraction reaches the colon and is catabolized by the intestinal microbiome [51]. Tryptophan metabolites are known to be bioactive compounds that play important roles in cancer and immune regulation. Li et al. reported that kynurenine, a product of tryptophan catabolism, was the most significantly upregulated serum metabolite in response to anti-PD-1 antibody and an increased serum kynurenine/tryptophan ratio was associated with worse overall survival in melanoma and renal cell carcinoma patients [52]. Recently, Karayama et al. examined plasma tryptophan metabolites in 19 patients with non-small cell lung cancer treated with ICIs and found that low levels of 3-hydrozyanthranilic acid was significantly associated with longer median progression-free survival in patients with non-small cell lung cancer [53].

It also has emerged that the purine nucleoside inosine produced by the intestinal microbiome is associated with ICI therapy responses. In a preclinical model, Tanoue et al. demonstrated that GF mice inoculated with an 11-member bacterial consortium had increased IFNγ positive CD8^+^ T cells and enhanced anti-tumor responses. A metabolic analysis found increased levels of several molecules, including mevalonate, dimethylglycine and inosine, in both cecal contents and the serum of GF mice inoculated with the consortium, compared to GF mice [29]. What role these metabolites may play were not fully evaluated in this study. Recently, Mager et al. identified a high abundance of the purine metabolite inosine in the serum and cecal contents of *Bifidobacterium pseudolongum*-monocolonized GF mice who exhibited enhanced anti-tumor responses by ICIs [54]. They further found that mice administered with inosine showed improved anti-tumor effects from anti-CTLA-4 antibody therapy, a benefit that was dependent on A_2A_R signaling specifically in T cells.

In contrast, some metabolites have been reported to have detrimental effects on ICI efficacy. Fecal levels of organic compounds such as 2-pentanone (ketone) and tridecane were associated with early progression of tumor in 11 non-small lung cell carcinoma patients treated with anti-PD-1 antibody [49]. In another study, non-small cell lung cancer patients with primary ICI resistance showed significantly increased serum indoleamine-2,3-diozygenase (IDO) at the first follow-up scan compared to baseline, suggesting IDO metabolism can play an important role in ICI resistance [55].

Together, these clinical studies and preclinical experiments indicate that not only the commensal bacteria but also microbial-derived metabolites may be important in impacting on the efficacy of ICIs (Table 1). Thus, characterizing and quantifying the profile of microbe-derived metabolites could be a means to predict and induce effective response to ICIs. However, our understanding of the microbial metabolome and its interactions with carcinogenesis and anti-cancer treatments is still in its infancy. Further larger follow-up clinical studies and systematic studies of larger panel of metabolites will help to confirm and expand upon these associations.

**Table 1:** The microbial-derived metabolites and ICI responses in clinical studies

Metabolites	Patient (n)	Disease	ICIs	Effects	References
SCFAs • Fecal acetate (high) • Fecal propionate (high) • Fecal butyrate (high) • Fecal valeric acid (high) • Plasma isovaleric acid (high)	52	Solid cancer tumor	Anti-PD-1	Longer PFS	[48]
SCFAs • Fecal propionate (high) • Fecal butyrate (high) Fecal lysine (high) Fecal nicotinic acid (high)	11	NSCLC	Anti-PD-1	Longer responses	[49]
SCFAs • Plasma butyrate (high)	40	Metastatic melanoma Metastatic prostate carcinoma	Anti-CTLA-4	Shorter PFS	[50]
SCFAs • Plasma propionate (high)	45	Metastatic melanoma	Anti-CTLA-4	Shorter PFS	[50]
Serum kynurenine/tryptophan ratio (high)	106	Advanced melanoma Advanced RCC	Anti-PD-1	Shorter OS	[52]
3-Hydrozyanthranilic acid (low)	19	NSCLC		Longer PFS	[53]
Fecal 2-pentanone (high) Fecal tridecane (high)	11	NSCLC	Anti-PD-1	Early progression	[49]
Serum IDO	23	NSCLC	Anti-PD-1	ICI resistance	[55]

ICI, immune checkpoint inhibitor; IDO, indoleamine-2,3-diozygenase; NSCLC, non-small cell lung cancer; OS, overall survival; PFS, progression-free survival; RCC, renal cell carcinoma

### Intestinal microbiome and risk of ICI-associated colitis

ICIs are associated with increased T cell activation and effective anti-tumor immune responses in a subset of patients. However, this treatment in some patients can also trigger serious immune-related adverse effects [7]. One of the most common serious adverse events is ICI-associated colitis [8]. ICI-associated colitis must be recognized quickly as it requires interruption of ICI therapy and appropriate treatment [7]. Multiple pathogenesis factors of ICI-associated colitis have been proposed, including microbiome dysbiosis [56, 57]. In one study, Vetizou et al. found alterations in the mucosal barrier following administration of anti-CTLA-4 antibody in mice, consistent with subclinical colitis [24]. This alteration of the mucosal barrier was more apparent in SPF mice than in GF mice, suggested a role of commensal bacteria in anti-CTLA-4 antibody-induced pathology. This study also identified that specific bacterial strains, such as *Bacteroides fragilis* and *Burkholderia cepacia*, abrogated ICI-associated colitis in SPF mice via promoting the proliferation of inducible T cell co-stimulator (ICOS) positive regulatory T cells (Tregs) in the lamina propria. In another murine study using dextran sulfate sodium for induction of colitis, it was shown that *Bifidobacterium* could largely rescue mice treated with ICIs from immunopathology while preserving anti-tumor effects [58]. One limitation of murine models to study ICI-associated colitis, however, is that in the absence of chemical insults or genetic deficiencies, mice generally do not develop clinical colitis after ICI therapy, in contrast to patients treated with ICIs [24]. For that reason, there are few preclinical reports about associations between ICI-associated colitis and microbiome dysbiosis, and it has been remained unclear whether specific bacteria can contribute to ICI-associated colitis.

In humans, ICI-associated colitis is more common during CTLA-4 blockade than during PD-1/PD-L1 blockade [7]. Dubin et al. reported that increased representation of the phylum Bacteroidetes was associated with resistance to development of ICI-associated colitis in a study of metastatic melanoma patients treated with anti-CTLA-4 antibody [59], a finding that was later reproduced by another study [8]. In contrast, increases in bacteria from the phylum Firmicutes, including *Faecalibacterium prausnitzii* and *Gemmiger formicilis*, have been demonstrated to be associated with a higher incidence of colitis [12]. Interestingly, while treatment with antibiotics prior to ICIs has been associated with reduced response rates to ICI therapies, antibiotics do not seem to impact on the frequency or severity of immune-related adverse events [41].

Evidence for potential benefits of reconstituting the intestinal microbiome of patients by introducing a FMT from a healthy donor has been well-demonstrated in randomized clinical studies of other types of colitis, including recurrent *Clostridium difficile*-associated colitis and inflammatory bowel disease [60, 61]. In ICI-associated colitis, a case series of ICI-associated colitis treated with FMT has been reported [62]. These two patients were successfully treated with FMT from a healthy unrelated donor. There was no observable trend in α-diversity following FMT, though the number of observed operational taxonomic units was increased. Follow-up colonic biopsy samples from these two patients demonstrated improvements in the infitrating immune cell subsets, with a substantial decrease in CD8^+^ T cells in both patients and an increase in infiltrating Tregs in one patient. These data suggested that modulation of the intestinal microbiome with FMT may abrogate ICI-associated colitis. However, because of the limited sample size, further studies with larger cohorts are required to confirm FMT efficacy in ICI-associated colitis. Thus, the intestinal microbiome may be an attractive therapeutic target for the treatment and prevention of ICI-associated colitis. However, despite its possible efficacy, FMT therapy still remains a controversial treatment due to potential drawbacks, including the challengs of securing a healthy microbiome donor and ensuring that FMT products are free of potential pathogens.

### The association between microbiome and immune reactivity

**Fig. 1:**
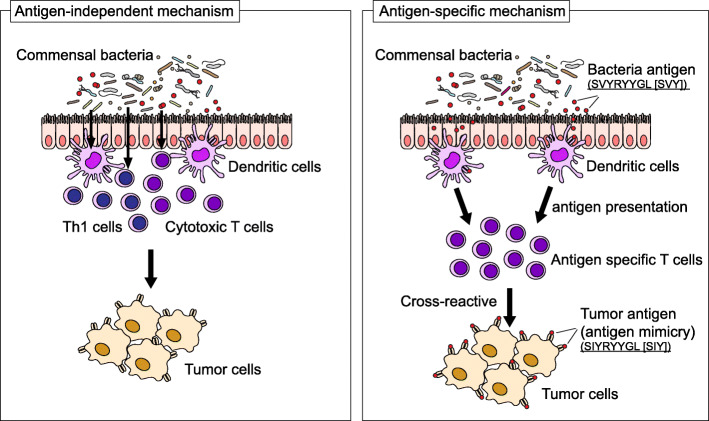
The intestinal microbiome may help determine outcomes of ICI therapy. Increased intestinal microbiome diversity and the presense of specific intestinal bacteria are associated with both responses and toxicity following ICI therapy. One possible group of mechanisms is antigen-independent, via induction of mucosal and systemic immune responses, especially Th1 and cytotoxic T cell responses, by the microbiome (left). Alternatively, antigen-specific mechanisms, specifically antigen mimicry between microbial and tumor antigens, could modulate anti-tumor immune responses. For example, T cell targeting an epitope SVYRYYGL (SVY) expressed in *Bifidobacterium breve* cross-react with a model neoantigen, SIYRYYGL (SIY) [63]. Antigen-specific T cells that can cross-react against both commensal bacterial and tumor antigens may play a role in ICI therapy (right)

Currently, mechanisms underlying modulation of ICI therapy by the intestinal microbiome remain unclear. A key mechanistic question is how certain microbial species can modulate immunity. Evidence has emerged that the intestinal microbiome can modulate the efficacy of ICIs via two general categories of mechanisms, including those that are antigen-independent and those that are antigen-specific (Fig. 1).

Researchers have found that differences in the composition of the intestinal microbiome can induce changes in mucosal and systemic immune responses. The differentiation of naïve CD4 T cells into various effector subsets, including T helper type (Th) 1, Th2, Th17, Tregs, and T follicular helper cells, has been particularly well-demonstrated to be modulated by microbes. Th1 responses can arise via activation of dendritic cells and cytotoxic T cell responses across the intestinal barrier [11, 25, 26, 28, 29, 40]. Effector Th1 and Th17 responses act to target and eliminate potential pathogens invading the host. In addition to adaptive immune cells such as T cells, the innate immune system can be modulated by microbial signals via a variety of innate signaling pathways, including toll-like receptors (TLRs), which play an important role in distinguishing commensal microbes from pathogenic organisms. Innate immunity involves various types of cells of the myeloid lineage, including dendritic cells (DCs) and macrophages, and innate lymphoid cells (ILCs), which include natural killer cells [64]. Strategies to target aspects of innate immunity can enhance ICI therapies. TLR7 and TLR9 agonists, when injected intratumorally, augmented effects of anti-PD-1 antibody in head and neck squamous cell carcinoma via immune cell activation [65]. In another study, *Lactobacillus rhamnosus GG*, one of the most well-characterized and used probiotics, promoted innate immune responses against tumor via activation of DCs when combined with anti-PD-1 antibody in a preclinical model [66]. Thus, via antigen-independent mechanisms, the intestinal microbiome or microbiome-derived ligands can modulate the efficacy of ICIs by engaging pathways expressed by adaptive and innate immune cells.

Another group of mechanisms can be termed antigen-specific, where antigen mimicry between microbial and tumor antigens can impact on anti-tumor immune responses. Neoantigens, known to arise from mutations in tumor cells as part of the process of carcinogenesis, can be targeted by T cells and are likely critical to successful eradication of tumors by ICI therapy [67, 68]. One study demonstrated that *Bifidobacterium breve*, a commensal intestinal bacterial species, could augment anti-tumor response by amplifying T cells recognizing a Bifidobacteria antigen with a similar epitope to that of a tumor antigen [63], indicating that antigen mimicry from intestinal microbes can influence T cells and augment a cross-reactive anti-tumor response. It remains to be seen how commonly antigen mimicry occurs and how impactful a role it can have on the robustness of anti-tumor effects, to clarify how the intestinal microbiome can lead to augmented anti-tumor effects in ICI therapy. A recent study identified major histocompatibility complex (MHC) class I-binding epitopes in the tail length tape measure protein (TMP) of a prophage found in the genome of the bacteriophage *Enterococcus hirae* and demonstrated that *Enterococcus hirae* harboring this prophage induced a TMP-specific MHC-restricted CD8^+^ T cell response upon immunotherapy with cyclophosphamide or anti-PD-1 antibodies in a mouse tumor model [69]. They also found an association between the presence of the Enterococcal prophage in stool, expression of a TMP-cross-reactive antigen by tumors, and long-term benefit of anti-PD-1 antibodies in renal and lung cancer patients [69]. These data suggested that this type of microbe-cancer cross-reactivity might be broadly clinically relevant. Further identification of homologous antigens found in microbes relevant to specific tumors as well as demonstration of T cell populations recognizing both microbial and tumor antigens will require additional studies.

## Conclusions

Intestinal microbes and microbial-derived metabolites can clearly have impactful effects on host immunity. Recent studies indicate that avoiding both an unfavorable microbiome as well as antibiotic-associated dysbiosis help to optimize ICI results, and a baseline intestinal microbiome assessment could be one approach to help predict responses associated with ICIs. Currently, clinical trials to elucidate the association between microbes, microbial-derived metabolites, and ICI efficacy or toxicity are ongoing (Table 2). Although the field includes findings with some inconsistencies across studies regarding which bacterial taxa are most associated with tumor responses in both preclinical and clinical studies, complementary assays to profile the microbiome including shotgun metagenomic sequencing and metabolomics, as well as characterizing the local tumor microbiome should further our understanding of how microbes are modulating tumor responses to ICI therapy.

**Table 2:** The ongoing clinical trials aimed to investigate the role of the intestinal microbiome modulation in ICI therapy

NCT number	Disease	Patient (n)	Brief study description	Primary endpoints	Enrollment status	Study phase
NCT04552418	Solid tumor	12	Pilot study of intestinal microbiome modification with potato starch supplement in cancer patients treated with a dual ICIs	Percentage of patients able to adhere to treatment Serious AEs	Not yet recruiting	1
NCT04107168	Melanoma Renal cancer Lung cancer	1800	Observational study to investigate how the microbiome correlates with efficacy and toxicity of ICIs	PFS	Recruiting	–
NCT03819296	Cutaneous melanoma Malignant genitourinary system neoplasm Malignant solid neoplasm Lung cancer Colitis	800	The study to evaluate the role of the intestinal microbiome and efficacy of FMT on ICI-associated GI complications	The intestinal microbiome Incidence of AEs of FMT	Recruiting	1/2
NCT04204434	Advanced cancer Neoplasms	150	The study to explore biomarkers for ICIs such as serum predictors, bacteria, or bacterial products in the intestinal microbiome	Serum predictors of response to ICIs	Recruiting	–
NCT04579978	Advanced solid tumor	60	The study to examine potential mechanisms by which gut bacteria in the intestinal microbiome impact on ICI response	The intestinal microbiome-associated ICI response The intestinal microbiome-associated ICI toxicity	Recruiting	–
NCT04038619	Colitis Diarrhea Malignant genitourinary system neoplasm	40	The trial to study how well FMT works in treating ICI-associated colitis	Incidence of FMT-related AEs Clinical response or remission of colitis	Recruiting	1
NCT04758507	Renal cell carcinoma	50	The study to evaluate the efficacy of targeted FMT	PFS	Recruiting	1/2
NCT04189679	Non-small cell lung cancer	60	The study to identify predictive metabolic, metagenomic, and immune signature of ICI response	The change of metabolic signature	Recruiting	–

AEs, adverse events; FMT, fecal microbiota transplantation; GI, Gastrointestinal; ICI, immune checkpoint inhibitor; PFS, progression-free survival

Thus, the composition of the intestinal microbiome may impact on not only the strength and durability of anti-tumor effects but also unfavorable intestinal inflammation in patients treated with ICIs. More studies are needed to achieve a better understanding of the interactions between the host and the intestinal microbiome, with a goal of identifying microbiome-based biomarkers of outcomes and designing strategies to target the microbiome to enhance the efficacy and safety of cancer immunotherapy.

## Data Availability

Not applicable

## Abbreviations

16S rRNA: 16S ribosomal RNA
CTLA-4: Anti-cytotoxic T lymphocyte antigen-4
DCs: Dendritic cells
GF: Germ-free
FMT: Fecal microbiota transplantation
GWAS: Genome-wide association studies
ICIs: Immune checkpoint inhibitors
ICOS: Inducible T cell co-stimulator
IDO: Indoleamine-2,3-diozygenase
IFNγ: Interferon-γ
ILCs: Innate lymphoid cells
MHC: Major histocompatibility complex
PD-1: Programmed death protein-1
PD-L1: Programmed death-ligand 1
SCFAs: Short-chain fatty acids
SPF: Specific-pathogen-free
Th: T helper type
TLRs: Toll-like receptors
TMP: Tape measure protein
Tregs: Regulatory T cells
